# Research on the Spatial Structure of the European Union’s Tourism Economy and Its Effects

**DOI:** 10.3390/ijerph18041389

**Published:** 2021-02-03

**Authors:** Wujie Xie, Haijian Li, Yufang Yin

**Affiliations:** 1 College of Business, Shanghai University of Finance and Economics, Shanghai 200433, China; yinyufang1@shnu.edu.cn; 2 School of History Culture and Tourism, Jiangsu Normal University, Xuzhou 221116, China; 6020100051@jsnu.edu.cn; 3 Tourism College, Shanghai Normal University, Shanghai 201418, China

**Keywords:** tourism economy, spatial structure, social network, the European Union

## Abstract

With the implementation of European integration policies such as the single market, the Euro and the Schengen Visa, the EU member states are developing closer economic ties through tourism, and their level of tourism integration is constantly improving. Taking the 28 EU member states as research objects, this paper constructs a tourism economic connection network among them, measures the strength of their tourism economic connections from 1995 to 2018 by using the modified gravity model and social network method, and analyzes the spatial structure characteristics and effects of the EU tourism economy. The results are as follows: (1) The tourism economic ties of EU member states are growing increasingly close, enhancing network stability. (2) Germany, France, Italy, Austria and the United Kingdom are the top five countries in the degree centrality and closeness centrality rankings, meaning that they are located in the center of the network and have great influence, and the network is becoming increasingly concentrated. Germany, Italy, Sweden, Austria and France play an important intermediary role in the network, and the centrality of most member states has increased. (3) The core areas are mainly concentrated in Western Europe, Southern Europe, Mediterranean mainland countries and Central Europe, while the marginal areas are mainly concentrated in Eastern Europe, Northern Europe and Mediterranean island countries; the network connection density of the core area, the network connection density of the marginal area, and the network connection density between the core and marginal area overall show an increasing trend. (4) Improvements in the complete network connectedness and a reduction in graph efficiency can significantly reduce differences in EU tourism economic development levels and improve spatial equity.

## 1. Introduction

Regional integration forms a network space that is based on relationships and driven by national power. The multidimensional proximity within regional integration is a driving force of its further rapid development and the deep-seated reason for the diversification of its organizational forms. Regional integration reshapes the local development model and economic geographical space of member states. The process of regional integration is a multidimensional spatial phenomenon. Its complex development characteristics and multidimensional economic effects, as well as its deep construction mode and complex social impact, have practical significance as a reference and theoretical research value. The interwoven network of culture, cognition, system, economy, trade, society and environment within regional integration can diminish barriers to the liquidity of people, goods, capital and services. With the development of regional integration, the breadth and depth of social impact are also constantly extending, which has an important and profound impact on the formation and shaping of tourism networks characterized by the flows of people.

As a typical model of regional integration, the EU has been recognized as the best and largest economic policy experimental area in the world, affecting all levels of Europe. One of the most significant areas affected by integration is service trades, and tourism is an industry deeply and comprehensively affected by the integration process. The importance of tourism activities has been highlighted at the regional level and at the subregional and national levels. In addition, as an industry that can improve social and economic welfare, tourism has been implemented as a reliable tool to achieve the ultimate goal of integration and cohesion [1]. In the process of regional integration in Europe, the spillover and marginal effect of extensive regulation and neighboring policies has had a significant impact on the development of tourism-related industries [2]. Tourism also has a dual economic and social character, and non-tourism concerns (e.g., regulations over the European market as a whole or the creation of employment opportunities and economic activities in less developed areas) influence inbound and outbound policies. By the end of 1992, 76 of the 278 proposals to establish a unified market in Europe had an impact on tourism. EU policies aimed to enhance the European single market and support industrial competitiveness, sustainable innovation and entrepreneurship. This inevitably led to the emergence of new policies in the field of tourism and promoted the development of regional tourism economic networks in the EU. Due to differences in the timing of accession, economic development level, tourism resource endowment, spatial distance and other factors, there is spatial differentiation in the EU tourism economy, and the tourism network is complex and hierarchical.

Against a background in which globalization and regional integration are affecting the world economy, global tourism has entered the stage of regional tourism spatial competition. This paper takes the EU tourism network as the research object, studies the spatial differentiation and evolution of the EU tourism economy, explores the EU as a world tourism destination participating in international competition, and investigates the construction of destination network sequences. The theoretical value and practical significance of this paper are reflected in the following two areas: (1) analyzing the tourism-related network across countries and exploring its laws at the social network level and (2) analyzing the characteristics and evolution process of the tourism economic network of EU member states, measuring the effect of the tourism economic network structure on EU tourism economic development, clarifying the positioning and role of each member state, and making recommendations to promote regional tourism cooperation and improve the specialization level of the tourism industry.

This paper consists of six parts. The first part introduces the research background. The second part offers the literature review, summarizing the achievements of scholars using the social network method to study the spatial structure of the tourism economy. The third part describes the research methods and data sources and introduces the revised tourism economic gravity model and social network method. The fourth part provides the empirical study: the complete network analysis includes the network spatial structure, network density and connectedness, while the individual network analysis includes degree centrality, betweenness centrality and closeness centrality. The core periphery model is used to analyze the spatial structure. The fifth part measures the effect of the tourism economic spatial network on tourism economic specialization and on the comprehensive tourism development level. The sixth part is the conclusion and insights.

## 2. Literature Review

For the study of European tourism’s spatial structure, more scholars focus on tourism planning, regional policy, and empirical model methods such as the spatial economic model and theories of evolutionary economic geography. This paper attempts to use the social network method to analyze the characteristics, evolution process and network effect of European tourism’s spatial structure. Therefore, this paper reviews the research on European tourism’s spatial structure and the application of social network theory to this.

### 2.1. Research on Spatial Structure Studies of European Tourism

In terms of policy effect on tourism, Jarvis and Kallas (2008) analyzed the impact of EU accession on the development trajectory of Estonia’s tourism industry [3]. Hall (2008) emphasized the need to interrogate a number of issues flowing from the policy neglect of tourism within the institutions of the EU [4]. In terms of marketing and planning, Valls et al. (2014) identified Europeans’ attitudes towards and preferences for European cities, furnishing useful data for city strategic planning to boost competitiveness [5]. Frantz (2018) thought that Vienna’s controversial political emergence contributed to constructing this cultural district as a new interpretation of the historic city [6]. With the popularity of all-inclusive holidays, Wall-Reinius et al. (2019) made an explorative study of Scandinavian tour operators and found that the local setting of the all-inclusive holiday was in fact a secondary consideration compared to the services and facilities on offer [7].

In urban and regional tourism, Amelung and Viner (2006) found that the climatic attractiveness of the Mediterranean region could have major impacts on the sustainability and distribution of tourism [8]. Rátz, Smith et al. (2008) examined the process of spatial transformation and regeneration in Budapest since 1989 [9]. Matoga and Pawłowska (2018) studied off-the-beaten-track tourism in the city of Krakow, Poland [10]. Taking Berlin, Paris, Vienna, Barcelona, Lisbon, Prague, Budapest, and Skopje as examples, Shoval (2018) et al. investigated the interrelationship between urban planning and tourism and its evolvement and transition over time [11]. Mansilla and Milano (2019) took the ethnographical approach to study the process that Barcelona had undergone, from a tourist city to a metropolitan tourist destination [12]. Bohlin, Brandt and Elbe (2020) analyzed concentration and the urban supremacy of four regions in central Sweden [13]. González-Pérez (2020) examined tourism gentrification in the historic center of Palma, Spain, through the increase in luxury hotels and short-stay holiday home rentals [14].

Apart from social network analysis, there are many other methods to study tourism spatial patterns. Paci and Marrocu (2014) used a spatial growth regression framework to analyze the impact of domestic and international tourism for 179 European regions [15]. Evolutionary economic geography (EEG) applied to tourism (with existing cases from the highly developed countries of Canada, Sweden, Denmark, Italy and Australia) is an emerging sub-field of research within economic geography and is still developing (Brouder 2014) [16]. Truchet et al. (2016) analyzed how tourist attractions influence tourism development, using count data models and an econometric analysis [17]. Soszyński et al. (2018) used cartographic spatial analysis and semi-structured interviews with community residents to study 17 tourist villages in the Łeczna-Włodawa Lake District of eastern Poland [18]. Romão and Nijkamp (2019) analyzed the impacts of innovation, productivity and specialization on tourism competitiveness within 237 European regions over 8 years, using spatial econometric analysis [19]. Mansilla and Milano (2019) took the ethnographical approach to study the process of Barcelona’s tourism. Spatial cross-regressive models are estimated using the System Generalized Method of Moments to explore the implications of tourism for the regional cohesion of the European Union (Llorca-Rodríguez et al. 2020) [20].

In the 1960s, researchers began to incorporate mathematical developments into the study of social relations. Later, this technology expanded beyond social fields (Scott 1991) [21]. The study of networks has undergone extensive development over the last three decades in various social and organizational fields (Zaheer et al. 2010) [22]. The core concept of SNA (social network analysis) is to analyze social phenomena from the perspective of a ‘network’ (Asero et al. 2016; Casanueva et al. 2016). Morrison et al. (2004) considered tourism development as the successful working of organizational alignment in the form of partnerships or networks [23]. In the 1990s, the social network method was applied to tourism research, providing rich technical indicators for measuring the spatial structure of tourism destinations; analyzing the characteristics of tourism’s spatial structure, evolution process and network effects; and providing a reference for tourism destination cooperation and spatial optimization, being regarded as an excellent paradigm for tourism spatial research (Scott 2008) [24]. Research on the spatial structure of tourism destinations from the perspective of social networks focuses on spatial network characteristics and evolutionary characteristics among scenic spots within the destinations on the one hand, and on the structural characteristics, evolutionary process, development mode and influencing factors of the spatial network among destinations on the other. Some authors have used the network method to study the field of tourism research networks (Baggio et al. 2010; Racherla and Hu 2010; Albrecht 2013; Benckendorff and Zehrer 2013; Casanueva et al. 2016) [25,26,27,28,29] and to conduct tourism keyword and citation analysis (Benckendorff 2009) [30], creating a more comprehensive understanding of this method’s applications in tourism research. Zhang et al. (2019) analyzed 67 papers on social network analysis, which were published in the top ten international tourism research journals from 2009 to 2018, from three aspects: research perspective and method, research content, and application of theory [31]. In user generated content travel research, Cervi (2019) studied the social networks in Italian online travelers’ community Ho sempre voglia di partire, and described the interactions that characterized the online community [32].

### 2.2. Research on the Network Structure of Scenic Spots in Tourism Destination

Pavlovich (2003) discussed the evolution and transformation process of tourism destinations, especially the evolution of service facilities, with the case of the Whitmore Cave scenic area in New Zealand. This study found that the higher the density of the network, the stronger the cohesion of the destination [33]. Saxena (2005) constructed a relational framework using principles of relationship marketing and the networks approach to examine the nature of exchange structure in three case study areas located in the Peak District National Park (PDNP) in the United Kingdom [34]. Baggio (2008) used the network method to study the evolution process of tourism destination networks [35]. By constructing a network of scenic spots in Baltimore, Maryland, and evaluating their value and connecting paths, Stienmetz and Fesenmaier (2015) pointed out that the process of destination value creation can be conceptualized as a network formed by the interaction between tourists and destinations [36]. Martínez-Pérez1 and Beauchesne (2018) analyzed the effects of closed networks and diverse networks on firm innovation using a sample of 215 hospitality and tourism firms located in the World Heritage Cities of Spain, and found an inverted-U-shaped relationship between closed networks and firm innovation [37]. Kang et al. (2018) analyzed the spatial structure of the Seoul tourist attraction system based on social network analysis technology using spatial statistics and found that a tourism destination can have multiple anchor points (i.e., scenic spots), and the spatial distribution pattern of the central area has a hierarchical structure and varies according to the length of stay [38]. Lee and Kim (2018) took shopping tourists as the research objects, constructed a spatial network of 28 scenic spots in Seoul’s Capital District, and evaluated their centrality and spatial structure changes [39]. Brandão et al. (2019) took the coastal region of Aveiro as an example, analyzing the innovation performance and network innovation processes of tourism firms located in coastal destinations [40]. Valeri and Baggio (2020) conducted a structural analysis of the network of Italian travel agencies and highlighted its self-organization characteristics that led to the development of informal communities [41]. Interorganizational networks are also important to tourism destinations (Baggio et al. 2010; Haugland et al. 2011; Tinsley and Lynch 2001) [25,42,43]. By employing a dyadic level of analysis and examining 990 dyadic observations from a Norwegian region of winter sports destinations, Aarstad et al. (2020) found that central organizations’ cobranding increases other less-central organizations’ cobranding through direct or indirect collaboration [44].

Liu et al. (2013) analyzed the centrality of the spatial network of 4A and 5A scenic spots in Xinjiang, explored the internal mechanism for the formation of tourism routes, and proposed a series of suggestions for tourism route arrangement [45]. Ju and Tao (2016) used the social network analysis method and GIS spatial analysis technology to analyze the spatial network structure of specialized happy farmhouse villages in Nanjing and revealed their spatial centrality [46].

### 2.3. Research on Network Structure among Tourism Destinations Based on Counties and Provinces

Based on different spatial scales, this paper studies the spatial structure of the tourism economy. Studies are conducted at the county and provincial levels. Shih (2006) took 16 self-driving camps in Nantou, Taiwan, as the research object, analyzed the network structure characteristics of degree centrality, betweenness centrality and structural holes in tourism destinations, and found that there were structural loopholes in the Nantou self-driving destination network and that relevant resource allocation should be strengthened [47]. Daskalopoulou and Petrou (2009) found that networks and entrepreneurial perceptions of a city’s asset base constituted important determinants of the successful operation of tourism businesses located in Patras, Greece [48]. Lee et al. (2013) used GIS and network methods to evaluate the centrality of 43 villages in Jangheung-gun and Jeollanam-do in South Korea, explored the role of centrality indicators in tourism integrated management, and found that core villages could provide more effective services [49]. D’Agata et al. (2013) constructed a spatial network of tourism destinations in Sicily, measured the degree centrality, betweenness centrality and closeness centrality, and used agglomerative subgroups to classify tourism destinations [50]. Borodako and Rudnicki (2014) conducted a network analysis of the air and train connections between selected cities in Central and Eastern Europe in the context of business tourism and identified the leaders of the industry in that region [51]. Based on a sample survey of Sicilian tourists, Asero et al. (2016) constructed a spatial network of tourism destinations and found that the mobility of tourists affected its shape, dimension and structure [52]. Kádár and Gede (2021) used network and cluster analysis of tourism flows mapped from user-generated Big Data to show how the Danube region is composed of different clusters of destinations, and how national boundaries have a strong shielding effect on the interregional movements of tourists [53]. Zhou (2008), Fang and Zhu (2014), Guo and Li (2014), Yu et al. (2015), Zhou and Xu (2015), Yan et al. (2018), Dong et al. (2018) and Wang et al. (2020) took the Chinese provinces of Sichuan [54], Hunan [55], Yunnan [56], Heilongjiang [57], Hunan [58], Hainan [59] and Xinjiang [60,61] as examples, respectively, analyzing the spatial structure, evolution process and role of the urban tourism economic network and exploring the spatial development model.

### 2.4. Research on Network Structure among Tourism Destinations Based on Countries and Regions

Studies also exist examining tourism economic networks at the national level or regional spatial level. Hwang et al. (2006) analyzed the multicity travel mode and network structure of American international tourists, discussed the role and status of each city among tourists in different regions, and proposed the bundling sales model of tourism destinations [62]. Bhat and Milne (2008) investigated the embeddedness, density and centrality of the destinations’ marketing network in New Zealand and found that the centrality of actors and the characteristics of the complete network structure affect cooperative marketing [63]. Scott et al. (2008) conducted a comparative study on the network structure and network cohesion of four different types of destination in Australia by means of centrality and other standards and observed that, the higher the degree of industrialization and the larger the scale of the network, the stronger the cohesion of the destinations [64]. García-Palomares et al. (2015) used the social network method, photo sharing and GIS spatial statistics technology to evaluate the spatial network characteristics of scenic spots in eight hotspot tourist cities in Europe. Their research shows that the concentration and dispersion of photos of each city and its main hotspots and the spatial concentration of tourists’ and residents’ photos are high, with significant differences [65]. Using the UK tourism industry data, Kim et al. (2021) revealed the significant effects of agglomeration economies on productivity within a specific region, and the significant spatial spillover effects across neighboring regions [66]. Through case studies of projects in Finland, Åland, Sweden and the Baltic region that were partly funded by the EU, Lemmetyinen (2010) found that the influence of EU funding has generated a network of interactive and learning destinations [67]. Lyócsa et al. (2019) studied the interconnectedness of changes in international tourism demand among 30 European countries within networks, finding that mutual interconnectedness was a typical feature of international tourism demand networks, and that countries with less developed (important) tourism sectors tended to be more interconnected with the international tourism demand of other countries [68]. Using international tourism data for 124 countries between 2000 and 2013, Chung et al. (2020) integrated cluster analyses and social network models and the results indicated that global tourism networks have become highly consolidated over time and that reduced transaction costs (e.g., language, distance, and visa policies) are more important in attracting international tourists than natural and cultural attractions [69].

Other relevant studies have been performed. Fang et al. (2013), Wang et al. (2013), Zhang et al. (2013), Ruan and Zheng (2017), Zou et al. (2017), Yang et al. (2018), and Shi et al. (2018), respectively took the Yangtze River Delta [70], Bohai Rim [71], Taihu Lake area [72], Pearl River Delta [73], the Economic Zone on the West Coast of the Taiwan Straits [74], Beijing-Tianjin-Hebei-Xiong’an Region [75], and Northeast China [76] as research objects, analyzing the characteristics, evolution process and mechanism of the urban tourism economic spatial network and exploring the regional development model. Zhu et al. (2012) and Wang et al. (2015) analyzed the evolution characteristics and influencing factors of the tourism economic network in the Yangtze River Delta Metropolitan Area [77] and Wuhan City Circle [78]. Yu et al. (2014), Liu (2015), and Wang and Gao (2019) analyzed the spatial structure, evolution process and spatial development mode of the tourism economic network of the Jianghuai urban agglomeration [79], the urban agglomeration in Central China [80], and the urban agglomeration in the middle reaches of the Yangtze River [81]. Ma and Long (2017) studied the spatial correlation of the economic growth of inbound tourism in China based on social network analysis [82]. Wang and Xia (2018) analyzed the spatial network structure and influencing factors of China’s provincial tourism economy [83].

Scholars use the social network method to analyze the network structure of tourism destinations at different spatial scales and reveal the characteristics of the complete network and the ego network. The literature review shows that: (1) on the spatial scale, the research areas range from single cities to urban agglomerations, metropolitan areas and large regions, but research focuses more on the urban tourism economic networks within a country and less on the study of international tourism economic networks; (2) most studies are based on static structure analysis, and fewer adopt dynamic evolution research; more attention is given to the structural characteristics of the tourism spatial network at a certain time point, and the dynamic evolution, formation mechanism and influencing factors of the tourism destination network are less commonly studied; (3) most studies focus on the description of tourism spatial network characteristics and pay less attention to the effect of network structure on tourism economic development.

Based on the above analysis, this paper uses the tourism economic data of 28 EU Member States (In 2017, the UK started the Brexit process and withdrew from the EU on 31 January 2020. From 1995 to 2018, the UK was a full member of the EU. Therefore, this paper studies the 28 member states of the regional EU, including Austria, Belgium, Bulgaria, Croatia, Cyprus, Czech Republic, Denmark, Estonia, Finland, France, Germany, Greece, Hungary, Ireland, Italy, Latvia, Lithuania, Luxembourg, Malta, the Netherlands, Poland, Portugal, Romania, Slovakia, Slovenia, Spain, Sweden, and the United Kingdom) from 1995 to 2018, calculates the tourism economic connection based on the modified gravity model, constructs an EU tourism economic spatial network, and analyzes its structural characteristics and effects with the help of the social network method. The overall characteristics and evolution trend of the tourism economic spatial network are reflected through network density, connectedness, graph hierarchy, and graph efficiency. The status and role of countries in tourism economic spatial networks are analyzed through degree centrality, betweenness centrality and closeness centrality. The network spatial pattern is characterized by the core periphery model. This paper uses panel data regression to measure the effects of the complete and ego network indicators of the tourism economic spatial network on spatial differences in tourism industry specialization and the tourism economic development level.

## 3. Methodology

### 3.1. The Modified Tourism Gravity Model

The gravity model, originating from Newton’s law of universal gravitation, was first applied to the study of international trade by Crampon (1966), who used “tourism flow” instead of “trade flow” and proposed a typical tourism gravity model. Then, some scholars adjusted the model adaptively by increasing or decreasing variables and controlling for time effect to predict tourism demand (Park and Jang 2014; Porto and Garbero 2018) [84,85].With the widespread application of the social network method in tourism research and the integration of research methods, social network research methods can be used to predict tourism demand, and the gravity model can be modified to build the basic data of the social network in order to study social network structure and conduct effect analysis (Dong et al. 2018; Yuan 2020) [60,86]. The modified gravity model, which is derived by measuring the economic connection between cities, is used to construct the asymmetric gravity matrix, through the mathematical model, for further social network analysis (Wang et al. 2006) [87]. The modified gravity model applied to social networks has experienced adjustment of explanatory variables and coefficients. The two variables of tourist arrivals and tourism receipts and the coefficient of tourism resources are widely used (Yang et al. 2018; Yan et al. 2018; Wang and Xia 2018) [59,75,83].

In this paper, the modified gravity model is used to build tourism connection strength, and tourism receipts, tourist arrivals and tourism resources are selected to reflect the mutual attraction between tourism destinations. The tourism receipts and tourist arrivals indicators represent the size and scale of tourism to a destination. These two indicators are comprehensive quality indicators of the tourism attraction of a destination, and are widely used and recognized in the literature. Additionally, it has also been found that tourism destinations with typical cultural or natural elements constitute some of the chief attractions for international tourists. Since cultural or natural attractions lead to increased tourism demand, it could be argued that those attractions that are officially authenticated, i.e., inscribed on the list of World Heritage Sites (WHSs) by UNESCO, should be relatively appealing to international tourists.

The gravity model of economic links among cities, regions and countries is modified according to the characteristics of tourism economic ties [74,82,88]. The indexes of population and GDP are replaced by international tourist arrivals and international tourism receipts. The relationship coefficient *k_ij_* is introduced [60,83], as in Formula (1). The modified tourism gravity model is used to measure the strength of tourism economic links between countries.

(1) $$F_{ij}=k_{ij}\frac{\sqrt{P_{i}*G_{i}}*\sqrt{P_{j}*G_{j}}}{D_{ij}^{2}},\;(k_{ij}=\frac{R_{i}}{R_{i}+R_{j}})$$

In Formula (1), *F_ij_* is the tourism economic connection strength between country *i* and country *j*. *P_i_* and *P_j_* are international tourist arrivals (10,000 people), and *G_i_* and *G_j_* are international tourism receipts (million US dollars). *D_ij_* is the distance between the capitals of country *i* and *j* (km), using the spatial friction index 2 commonly used by scholars. *R* is the number of world-class tourism resources, including world heritage sites, wetlands of international importance (Ramsar sites), and United Nations Educational, Scientific and Cultural Organization (UNESCO) Man and Biosphere Reserves. Taking tourism resources as the coefficient, this paper constructs an asymmetric assignment matrix of the tourism economic links of the EU Member States. To calculate tourism economic connection strength, the tourism economic connection strength of a country to other countries in the region is summed to obtain coefficient *F_i_* [74], as in Formula (2).

(2) $$F_{i}=\overset{n}{\underset{j=1}{\sum }}F_{ij}\;$$

### 3.2. Social Network Analysis

Social network analysis, based on the data of the “relation matrix”, provides a quantitative analysis of various relationships and reveals the relationship and structure between research objects.

#### 3.2.1. Complete Network Characteristic Index

Network density represents the degree of tourism economic proximity between network nodes. The nodes can be tourism stakeholders, destinations, enterprises or related groups in the social network analysis of the tourism field. The nodes in this paper are countries that are international tourism destinations.

The value range is (0, 1) and is calculated by dividing the number of actual relationships in the tourism economic network by the theoretical maximum number of relationships. The greater the network density, the closer the tourism economic ties, the greater the impact of the network structure on actors, and the more stable the network tends to be [60]. The connectedness degree reflects the robustness of the network. As for directed graphs, if any two points can be connected, it is called an association graph, and the degree of association of the network is measured by “accessibility”. If some points in the graph cannot reach each other, the graph shows less association. Graph hierarchy measures the degree to which nodes in a network are asymmetrically reachable. The more hierarchical the network is, the stricter the hierarchical structure between nodes, and more nodes are in subordinate and marginal positions in the network. Graph efficiency reflects the connection efficiency between network nodes. The more connections there are between nodes, the closer the network connection, and the lower the graph efficiency.

#### 3.2.2. Ego Network Characteristic Index

Centrality describes the central position of nodes in the network, including degree centrality, betweenness centrality and closeness centrality [89]. Degree centrality measures the central position of each node in the tourism economic network. This measurement is based on the number of nodes directly connected to the node without considering indirectly connected nodes. The more direct connections a node has with other members, the more central the node is in the network, and the more power it has. To compare the degree centrality of points in different graphs, Freeman (1979) proposed relative degree centrality, which is the ratio of the absolute centrality of a point to the maximum possible degree of a vertex. In a directed network, the calculation formula of the relative degree centrality of node *N_i_* is as follows:

(3) $$C_{RD}\left(i\right)=\frac{C_{DI}\left(i\right)+C_{DO}\left(i\right)}{2n-2}$$

In Formula (3), *C_RD_*(*i*) is the centrality of the relative degree of node *i*. *C_DI_*(*i*) is the entry degree of node *i*, which is the number of other nodes entering into the node, i.e., the number of direct relationships obtained by this node. *C_DO_*(*i*) is the out degree of node *i*, which is the number of relationships directly sent out by this node. *n* is the number of nodes.

Betweenness centrality is used to evaluate the control degree of nodes over various resources in the network. As the shortest intermediary between more countries, the higher the intermediate centrality of a country is, the more structural holes it has, and the stronger the control ability of nodes over network resources.

(4) $$C_{RB}\left(i\right)=\frac{2C_{AB}\left(i\right)}{\left(n-1\right)\left(n-2\right)}=\frac{2\sum_{j}^{n}\sum_{k}^{n}g_{jk}\left(i\right)/g_{jk}}{n^{2}-3n+2},\;(j\;\neq \;k\;\neq \;i,\;\mathrm{and}\;j<\;k)$$

In Formula (4), *C_AB_*(*i*) and *C_RB_*(*i*) are the absolute and relative betweenness centrality of node *i*, respectively. *g_jk_* is the number of shortest connection paths between *j* and *k*, while *g_jk_*(*i*) represents the number of shortest connection paths between *j* and *k* passing through node *i*.

Closeness centrality measures the extent to which a node is not controlled by other nodes. If the distance between a node and other nodes is very short, the node has a high degree of closeness centrality. Relative closeness centrality is used to compare the closeness centrality of points in different graphs. The larger the relative closeness centrality of a node, the greater the node’s core position in the network. [90].

(5) $$C_{RC}\left(i\right)=\frac{n-1}{C_{AC}^{-1}\left(i\right)}=\frac{n-1}{\sum_{j=1}^{n}d_{ij}}$$

In Formula (5), $C_{AC}^{-1}\left(i\right)$ and $C_{RC}\left(i\right)$ are the absolute and relative closeness centrality of node *i*, respectively, and *D_ij_* is the shortcut distance between nodes *i* and *j* (the number of lines included in the shortcut).

#### 3.2.3. Core-Periphery Model

The core periphery model shows which nodes are located in the core area and which are located in the peripheral area and reveals the internal relationship between the core and periphery. Through a diachronic cross-section comparison, the dynamic change process for the nodes in the core area and those in the peripheral area is analyzed. Using UCINET, the core area and peripheral area of the tourism economic network are analyzed.

### 3.3. Study Area

The EU is a unique economic and political union. The predecessor of the EU was the European Economic Community (EEC), whose founding members include Germany, France, Italy, Belgium, Luxembourg and the Netherlands. The EEC aimed to reduce unnecessary conflicts between countries and realize economic exchange and cooperation. With improvement in the level of economic integration and the expansion of cooperation areas, the pure economic union has evolved into an international organization that spans the policy fields of climate, environment, health, safety, justice and immigration. In 1993, a unified market for the free flow of goods, services, personnel and funds was realized, and the EEC was renamed the EU. Guided by the rule of law, the EU has achieved peace, stability and prosperity for more than half a century based on voluntary and democratic consultation among its member states.

The EU is rich in tourism resources. The number of world heritage sites, UNESCO Man and Biosphere Reserves, wetlands of international importance (Ramsar sites) and other tourism resources accounts for approximately half of the global total. With the advantages of a single market, the Schengen visa and a single currency, the EU has become one of the world’s important tourism destinations. In 2018, the EU received 562 million international inbound tourists, and international tourism receipts reached 480.8 billion US dollars, accounting for 40% and 33% of the global market, respectively [91]. According to the number of international inbound tourists and the income of international inbound tourism, five EU countries, France, Spain, Italy, the United Kingdom and Germany, all entered the top 10 tourism destinations in the world. The World Tourism Organization predicts that international tourist arrivals to the EU will reach 1.8 billion in 2030, and the EU will become the largest tourist destination (accounting for 41.1% of the global total).

Sources: World Tourism Organization (2018), European Union Tourism Trends, United Nations World Tourism Organization, Madrid.

### 3.4. Data Source

The data on international tourist arrivals and international tourism receipts of EU member states are derived from the World Development Indicators database of the World Bank. The spatial weight matrix calculates the distance matrix between countries according to the longitude and latitude of each capital. According to UNESCO, world-class tourism resources include world heritage sites (http://whc.unesco.org/en/list/), wetlands of international importance (Ramsar sites) (http://www.ramsar.org/sites-countries/ramsar-sites-around-the-world), and UNESCO Man and Biosphere Reserves (http://www.unesco.org/new/en/natural-sciences/environment/ecological-sciences/biosphere-reserves/europe-north-america/). The tourism resource coefficient is entered into the gravity model, and the asymmetric assignment matrix of EU tourism economic relationships is constructed. This paper uses UCINET6 to obtain the EU tourism economic network matrix, analyzes network density, network centrality and the core periphery model, and analyzes the characteristics and effects of the EU tourism economic network structure.

The time span of the data is from 1995 to 2018.

## 4. Spatial Structure Characteristics of the EU Tourism Economy

### 4.1. Analysis of Tourism Economic Connection Strength

Using the modified gravity model, the intensity of tourism economic links among EU member states is calculated, and the tourism economic ties of each member state are calculated according to Formulas (1) and (2) (as shown in Table 1). In terms of time, several time points that are of great significance to the development of the EU economy and tourism are selected: 1995, 2002, 2009 and 2018. In 1995, the EU’s Schengen Area was officially launched. The Schengen Agreement was signed by Germany, France, Netherlands, Belgium and Luxemburg in Schengen, Luxembourg, on 14 June 1985. The Convention entered into force in July 1995.The euro began to officially circulate in 2002. On 1 January 1999, the euro was officially issued within the scope of EU member states. It is a supranational currency with independent and legal status. According to the Maastricht Treaty, the euro was officially circulated on 1 January 2002. The Lisbon treaty came into force in 2009, which optimized the EU’s organizational structure and operational mechanism, improved operational efficiency and achieved closer cooperation. The Lisbon Treaty entered into force in 2009. Before this, it needed to be approved by all EU countries. After its entry into force, changes in the EU’s organizational structure and operational mechanism are reflected in three areas. First, the original practice of rotating the presidency of the EU Council was abolished and a permanent president of the EU Council was established, that is, the “President of the EU”, who represents the EU and makes public appearances on the international stage. The post has a term of two and a half years, and can be re-elected for one term. Second, the two posts of the representative for foreign affairs and security policy of the Council of the European Union and the member in charge of foreign relations affairs of the European Commission were merged to establish a new senior representative for foreign affairs and security policy of the EU, similar to a foreign minister, and the authority of the post was expanded, especially to include financial power over foreign aid. Third, some policy areas that should have adopted the principle of unanimous adoption were classified into the areas of majority voting. 2018 is the data cutoff year of this study.

The tourism economic connections of EU Member States are shown in Table 1. (1) From 1995 to 2018, the total number of tourism economic ties between countries showed a sustained growth trend, increasing by 407% from 4598.81 in 1995 to 23,342.20 in 2018. The tourism economic ties between countries showed an increasing trend, but there were spatial differences. Under the promotion of a series of policies, such as the Schengen Agreement, the Euro Zone and the Lisbon Treaty, EU integration was strengthened, and the development of tourism integration was promoted. Tourism economic ties gradually became closer, and the tourism economic ties between various countries showed an increasing trend. (2) The countries with higher and lower tourism economic ties are relatively stable in the ranking list. The top ranked countries include France, the United Kingdom, Austria, Germany and Italy, which are mainly concentrated in Western Europe. The countries at the bottom of the list include Luxembourg, Lithuania, Latvia, Malta, Cyprus, etc., and the reasons for their lower ranking are distance, time of EU accession and other factors. (3) The unbalanced pattern of regional tourism remains unchanged, and the development trend is improving. The intensity gap in tourism economic ties among EU member states shows a converging trend. From 1995 to 2018, the proportion of the top five countries in total tourism economic intensity decreased from 68.70% to 63.15%, while the proportion of the bottom five countries increased from 0.13% to 0.26%.

### 4.2. Characteristics of Complete Network

#### 4.2.1. Construction of Cyberspace Structure

**Figure 1:**
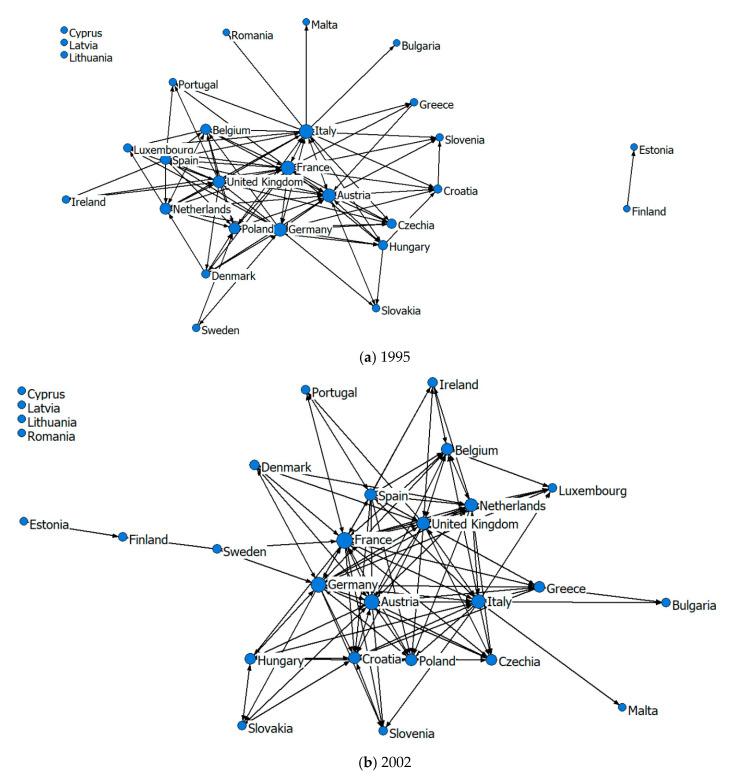

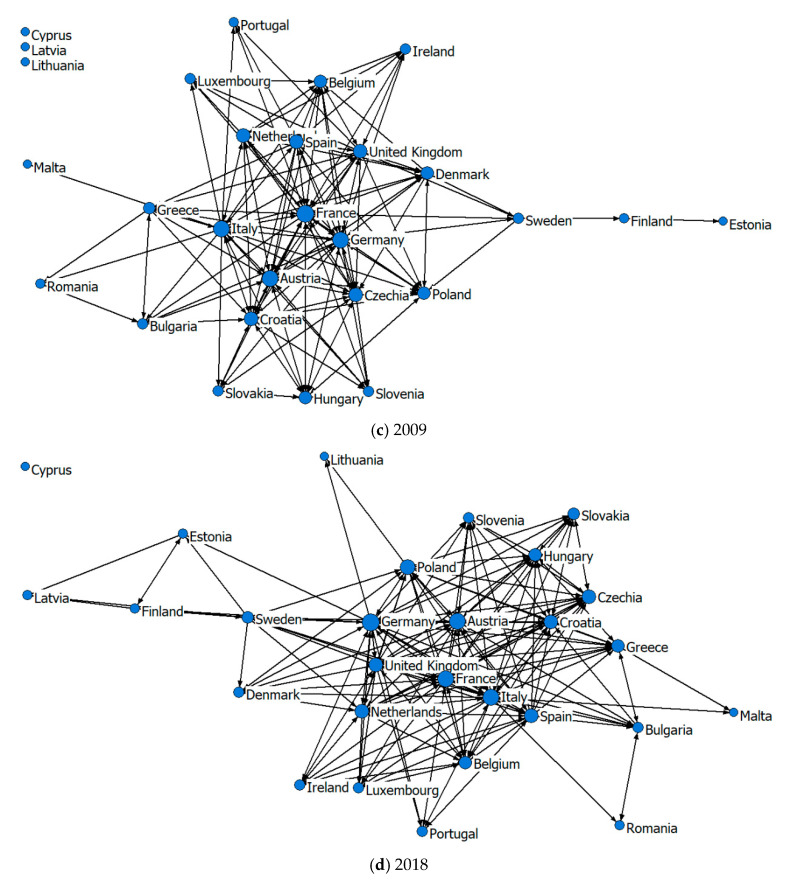
Tourism economic network in EU from 1995 to 2018.

According to the four initial 28 by 28 matrices of tourism economic connection intensity of the EU member states, the mean value of gravity after eliminating outliers is taken as the dividing value of the tourism economic network [92,93]. A value of tourism economic connection strength greater than or equal to the dividing value is recorded as 1, and that less than the dividing value is marked as 0. After binary processing of the initial matrix, the one-mode directed network of tourism economic links among EU member states is obtained, and the EU tourism economic connection network is constructed. The spatial structure of the EU tourism economic connection network is shown in Figure 1.

Figure 1 shows that in 1995, 2002, 2009 and 2018, the centers of the EU tourism economic network are France, Germany, Italy, Austria and the United Kingdom. Although the ranking list changed, these countries were all in the top five. In 1995, the outliers included Cyprus, Latvia and Lithuania. Romania became an isolated node in 2002 but not in 2009. In 2018, only Cyprus was isolated. On the whole, the structure of the EU tourism economic network is becoming increasingly complex, and tourism economic ties are growing increasingly close.

#### 4.2.2. Network Density

**Figure 2:**
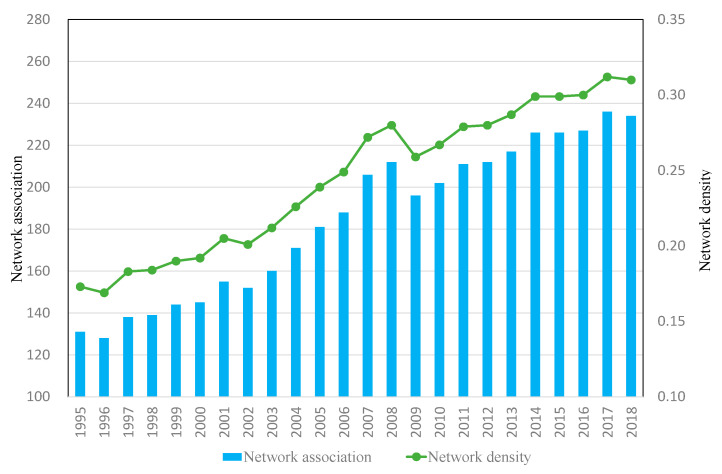
Network density and network association.

The higher the network density, the stronger the connection between nodes, and the more ways there are to achieve contact from other nodes, which is conducive to the development of each node. Figure 2 shows the evolution trend of spatial association and network density of the tourism economy in the 28 EU member states from 1995 to 2018. Spatial association increased annually, with the number increasing from 131 to 234. The complete network density also increased annually, from 0.17 to 0.31, indicating that tourism economic ties between EU member states were growing increasingly close. At the same time, 2008 can be seen as a cutoff point, as after 2008 the international economic crisis had a negative impact on tourism economic ties among EU member states.

#### 4.2.3. Network Relevance

**Figure 3:**
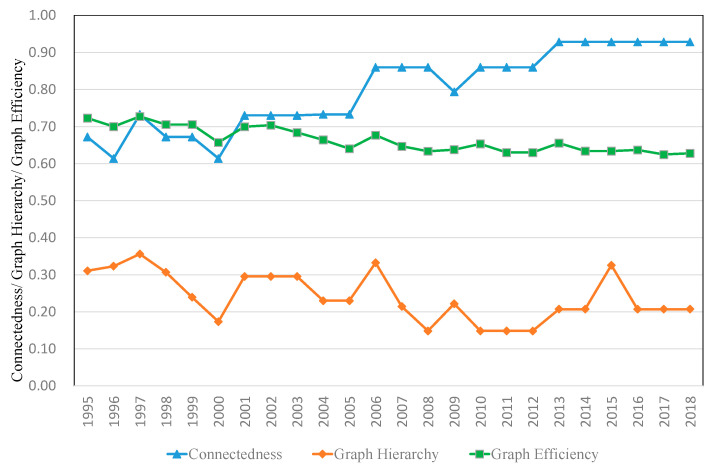
Network relevance.

The EU tourism economic network is measured by three indicators: connectedness, graph hierarchy and graph efficiency. Figure 3 shows that, from 1995 to 2018, connectedness n increased from 0.67 to 0.93, indicating that EU tourism economic ties were gradually growing closer, and there were very obvious spatial association and spillover effects. Graph hierarchy declined from 0.31 to 0.21, and the hierarchy of the spatial association structure was weak. The mutual connection and mutual influence of the tourism economy gradually strengthened. Graph efficiency showed an annual downward trend, from 0.72 to 0.63, indicating that the number of connections in the tourism economic spatial association network increased, and the stability of the network improved. From the measurement results of the three indicators, it can be seen that, with the development of the marketization process, the hierarchy system of the tourism economy weakened, spatial association increased, and the stability of the network was enhanced.

### 4.3. Characteristics of the Ego Network

The individual characteristics of EU member states in the tourism economic connection network are further analyzed. The degree centrality of EU member states in 1995, 2002, 2009 and 2018 is shown in Table 2. The betweenness centrality and closeness centrality of EU member states in 1995, 2002, 2009 and 2018 are shown in Table 3.

#### 4.3.1. Degree Centrality

(1) From 1995 to 2018, the degree centrality of EU member states showed a significant growth trend, and the number of isolated nodes decreased from three to one (as shown in Table 2 and Figure 1). Tourism economic ties between member states were gradually strengthened and showed a trend towards centralized development. The degree centrality of Germany, France, Italy, Austria and the United Kingdom increased significantly. Although the ranking changed, the five countries were always in the forefront, and their central positions were more prominent. In the spatial association network structure of the tourism economy, these five countries were in the central position in the network and had the most direct connections with other countries, holding greater resources and power. The degree centrality of Poland, the Netherlands, Croatia, Spain, the Czech Republic, Hungary, Greece and Belgium was higher than the average, and saw a large increase. Their tourism economic ties with other countries were strengthened, and their central positions were further improved. The lower ranking countries were Estonia, Romania, Malta, Latvia and Lithuania, which were in subordinate positions in the network. Among them, the tourism economic ties between Latvia, Lithuania and other member countries were strengthened, and they ceased to be isolated nodes. However, the degree centrality of Cyprus was 0 because it is located at the border of Asia and Europe, far from the tourist market of the European continent, and its tourism links with other EU countries were weak.

**Figure 4:**
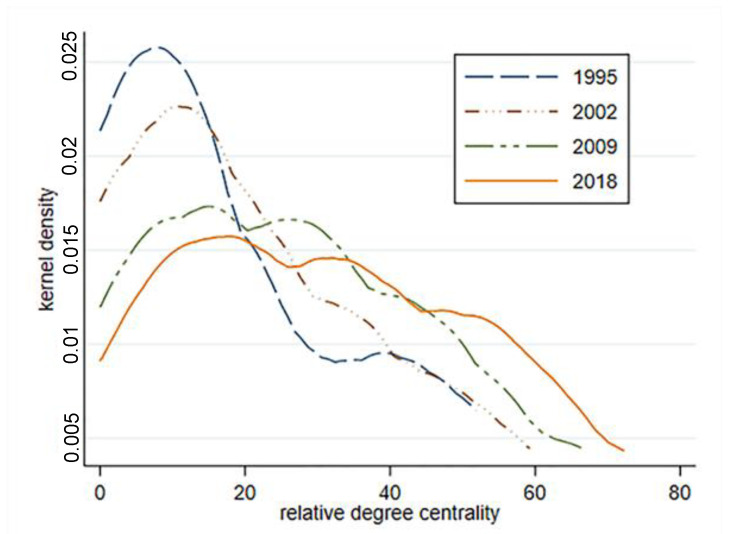
Kernel density estimation of degree centrality.

In Figure 4, the estimation results of the relative degree centrality kernel density of the EU tourism economic connection network from 1995 to 2018 show that the relative degree centrality kernel density estimation maps of the four periods are all right biased, indicating that the number of countries with more tourism economic ties is relatively small. The peak is getting lower and the right tail is getting longer, which reflects that the proportion of countries with fewer tourism economic ties is gradually decreasing, the overall distribution is shifting to the right, the number of countries with more tourism economic ties is increasing, and tourism economic ties are becoming closer.

(2) The out-degree analysis shows that the out-degree values of Germany, Italy, France, the United Kingdom, Spain, and Austria are large. These six countries are in the first camp, with a steady growth trend, showing that their status in the EU tourism economic network was continuously being consolidated, they had closer ties with other countries, and their tourism economic radiation capacity was growing increasingly prominent. The out-degree values of the Netherlands, Poland, the Czech Republic, Hungary, Croatia, Greece and Sweden are higher than the average (in 2018) and are in the second camp, showing rapid growth. Their tourism economic ties with other countries were gradually strengthened, and their radiation capacity was greatly improved. The out-degree values of Cyprus, Latvia, Lithuania and Malta are 0, indicating that the driving effect of these four countries on other countries was extremely weak, and they were relatively far from other countries in the EU.

(3) The in-degree values of Austria, France, Germany, Croatia, Italy, and Poland have rapidly grown, and the six countries rank at the top among EU member states. The in-degree values of the Czech Republic, the Netherlands, Belgium, Hungary, Greece and Slovenia are higher than the average (in 2018), and the six countries are in the second camp and affected by other countries. The in-degree value of Cyprus is 0. It is far from other central countries of the EU, so it is less affected by the tourism economy of other countries.

(4) The out-degree values of Germany, Italy, France, the United Kingdom and Spain are larger than their in-degree values, showing that the radiation impact of these five countries on other countries is greater than the radiation impact of other countries on them, and the five countries are more active in the core position of the tourism economic network. The in-degree values of Austria and Croatia are larger than their out-degree values, indicating that the radiation impact of other countries on these two countries is greater than their radiation impact on surrounding countries. The in-degree values of Poland are far larger than its out-degree values in 1995, 2002 and 2009 but almost the same in 2018, which led to a significant increase in the degree centrality of Poland.

(5) Degree centrality describes the level of tourism economic connection between nodes, while the centralization of the graph depicts the economic connection level of the whole network. The centralization of the graph reflects the degree of asymmetry and imbalance in the economic relations between nodes. The closer the centralization of the graph is to 100%, the more centralized the network. Table 2 shows that out-degree centrality has an upward trend in volatility, while in-degree centrality shows a downward trend, and out-degree centrality is greater than in-degree centrality. Relative degree centrality has a steady upward trend, rising from 37.18% to 44.44%. The EU tourism economic network concentration is strengthening, and it is unbalanced. Due to complementary tourism resources, spatial proximity, close economic ties and other reasons, tourism economic ties are mainly concentrated among certain countries. The links between tourism powers are increasing. Countries with radiation influence show a trend towards centralization, and the degree of imbalance increases. The countries receiving radiation from other nodes are increasing, showing a balanced trend.

#### 4.3.2. Betweenness Centrality

From 1995 to 2018, the betweenness centrality of most countries improved. Germany, Italy, Sweden, Austria, and France had high betweenness centrality, ranking in the top five, which indicated that these nodes had strong control ability and a supporting role for other countries, and they played an important intermediary role. They were at the core circle level in the EU, and their spatial relationships with other nodes were relatively close. The betweenness centrality of Sweden, Bulgaria, Finland, Greece, the Czech Republic, and Estonia passed 0, showing an upward trend and indicating that the intermediary ability of nodes to network resources was increasing. However, there were still 11 countries, including Denmark, Cyprus, and Ireland, whose betweenness centrality was 0, indicating that these countries were not intermediary nodes, had no ability to control resources, were placed in a marginal position and had weak tourism spatial connections. These countries had the characteristics of a small population, a small-scale tourism economy, and a long distance from the center of the European continent, which made it difficult for them to control and dominate other nodes in the network. Network centralization increased from 11.48% to 12.57%, showing a volatile upward trend.

#### 4.3.3. Closeness Centrality

From 1995 to 2018, the closeness centrality of each node country showed a significant growth trend, indicating that the links between regional nodes were strengthening and the regional influence of nodes was increasing. Although the rankings changed, the top five countries were always Germany, France, Italy, the United Kingdom and Austria, indicating that these five countries were closely connected with other countries. The top five countries had good accessibility and were less controlled by other nodes. They were in the center of the complete network and had the greatest influence in the region. Finland, Malta, Lithuania, Latvia and Cyprus were low-ranking countries. In these years, the number of isolated nodes reduced from three to one.

### 4.4. Core Periphery Analysis

**Figure 5:**
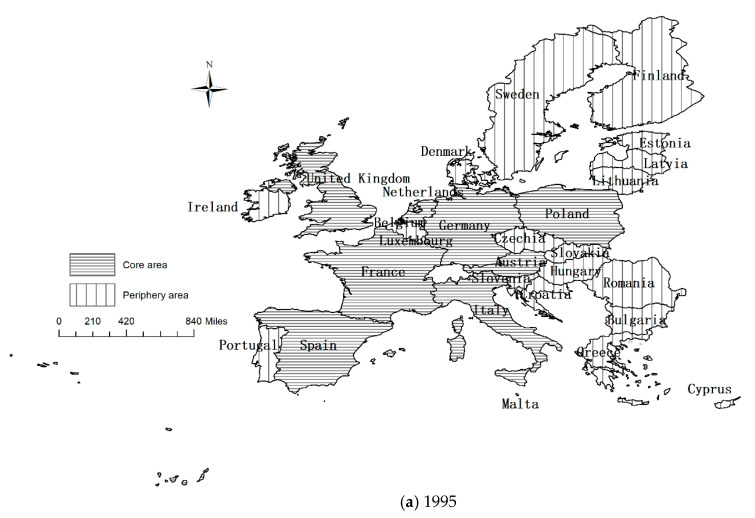

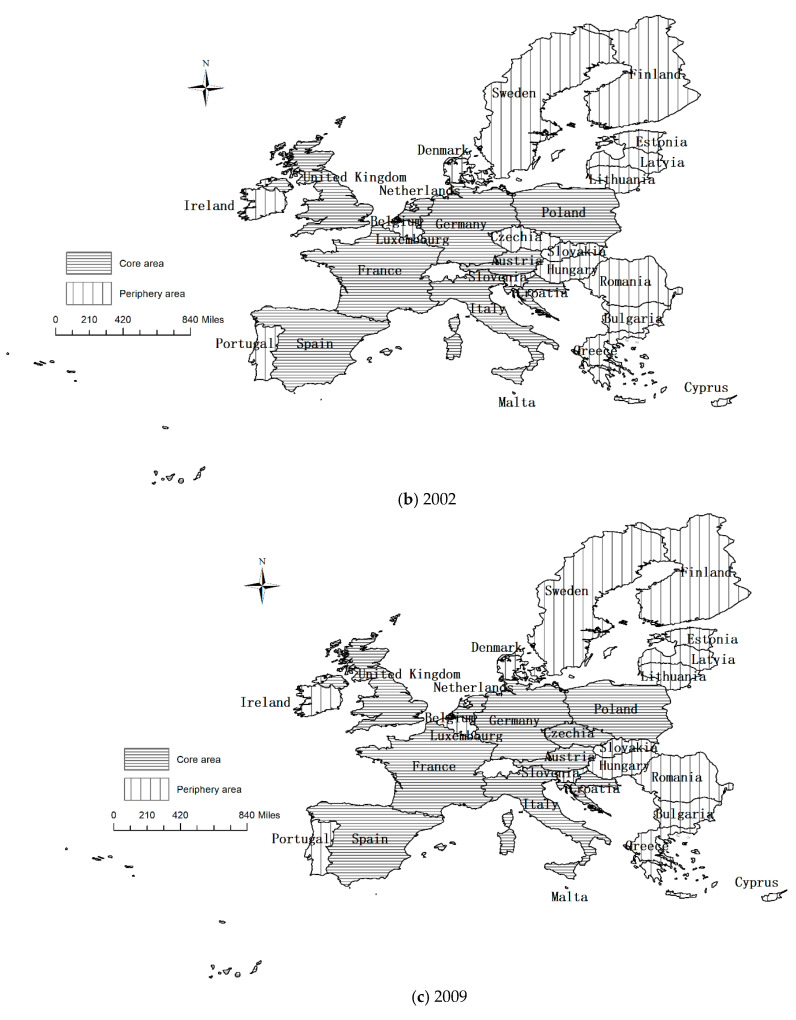

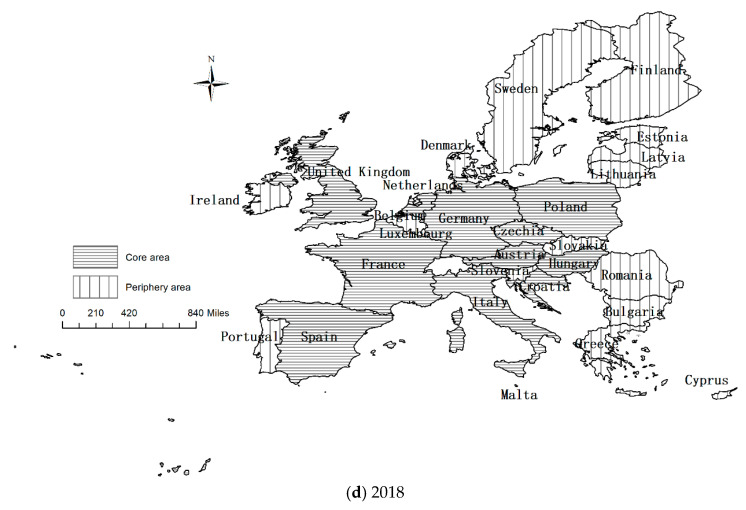
Core-periphery structure of the tourism economic network in the EU from 1995 to 2018.

According to the binary matrix of the EU tourism economic connection network, the core periphery analysis is carried out by using UCINET software. The EU member states are divided into core areas and peripheral areas, and the tourism economic connection density of the core area and peripheral area is calculated (in Figure 5).

From 1995 to 2018, the core area of the EU tourism economic connection network expanded. In 1995, the core area included eight countries: Austria, France, Germany, Italy, the Netherlands, Poland, Spain and the United Kingdom. In 2002, Croatia joined the core area, the Czech Republic joined in 2009, and Hungary in 2018. At that time, the number of core area countries had increased to 11, while the number of peripheral countries had decreased from 20 to 17. The 17 peripheral area countries were Belgium, Bulgaria, Cyprus, Denmark, Estonia, Finland, Greece, Ireland, Latvia, Lithuania, Luxemburg, Malta, Portugal, Romania, Slovakia, Slovenia and Sweden. In terms of space, the core areas were mainly concentrated in Western Europe, Southern Europe, the Mediterranean land area and Central Europe. Traditional world tourism destination countries are the absolute core areas of the EU tourism economy, while marginal areas were mainly concentrated in Eastern Europe and Northern Europe, as well as in Mediterranean island countries.

From the trend in the network density of the core peripheral structure of the EU tourism economy, it can be seen that the network density of the core area, peripheral area, and transitional zone between the core area and peripheral area showed different degrees of growth, which promoted the development of the complete network connection of the EU tourism economy. The network density among the core area members showed fluctuating development, declining from 0.911 in 1995 to 0.819 in 2002, and then increasing to 0.900 in 2018. Although there were fluctuations, the tourism economic ties of core area members were relatively close. From 1995 to 2018, the connection density of the core area and peripheral area increased from 0.306 to 0.406, which indicates that the tourism economic connection between the core area and peripheral area was strengthening. The network density of the peripheral area members increased from 0.013 to 0.063, but the tourism economic connection network of the peripheral area was not close.

### 4.5. EU Accession Will Effectively Enhance Degree Centrality

There were 12 countries that accessed the EU before 1995. After that, three countries accessed the EU in 1995, 10 countries in 2004, two countries in 2007 and one country in 2013 respectively (shown in Table 4). The accession to the EU can bring benefits of sharing the EU’s travel market, tourism resources and tourist facilities, as well as capital, technology and human resources, and gradually strengthening tourism economic ties with other EU member states. The degree centrality of countries has increased to varying degrees as they have become EU member states for different durations. Degree centrality is one of the important indicators of ego network characteristics, reflecting the central position of each country in the EU tourism economic network.

12 countries, including Germany, France, and Italy, accessed the EU before 1995. From 1995 to 2018, the degree centrality of these 12 countries was greatly improved. In 2018, the degree centrality exceeded the average value except for four countries: Denmark, Ireland, Luxembourg and Portugal. The centrality of Germany, France, Italy, Britain, the Netherlands and Spain has been continuously consolidated and placed at the central position of the network.

Austria, Sweden and Finland accessed the EU in 1995. Between 1995 and 2018, the degree centrality of Austria increased from 48.15 to 61.11, with a growth rate of 26.92%, and it ranked among the top five in the EU. The degree centrality of Sweden increased from 5.55 to 27.77, with a growth rate of 400%. The degree centrality of Finland increased from 1.85 to 11.11, with a growth rate of 500%. Ten countries including Poland, Czech Republic, and Hungary, accessed the EU in 2004. From 2004 to 2018, the degree centrality of Poland increased from 33.33 to 50, with a growth rate of 50%, and it ranked among the top five in the EU in 2018. The degree centrality of Czech Republic increased from 27.77 to 42.59, with a growth rate of 53%. The degree centrality of Hungary increased from 25.93 to 38.88, with a growth rate of 50%. The degree centrality of Czech Republic and Hungary exceeded the average value of degree centrality in 2018. The degree centrality of Slovakia increased from 12.96 to 24.07, with a growth rate of 85.71%. The degree centrality of Slovenia increased from 12.96 to 20.37, with a growth rate of 57.14%. Although the four countries of Estonia, Latvia, Lithuania and Malta rank low in degree centrality, tourism economic ties with other EU member states have shown a substantial increase and degree centrality has been increasing year by year since accession to the EU in 2004. Among them, the degree centrality of Estonia increased from 3.70 to 9.25, with a growth rate of 150%. Latvia (with accession in 2013) and Lithuania (with accession in 2004) broke away from being isolated nodes, and the degree centrality increased to 5.55 and 3.70, respectively, in 2018. The degree centrality of Malta increased from 1.85 to 3.70, with a growth rate of 100%. Bulgaria and Romania accessed the EU in 1995. From 2007 to 2018, the degree centrality of Bulgaria increased from 16.66 to 22.22, with a growth rate of 33.33%. The degree centrality of Romania increased from 11.11 to 16.66 in 2017, but dropped to 5.55 in 2018.

Croatia accessed the EU in 2013. The degree centrality of Croatia increased from 42.59 in 2013 to 46.29 in 2018, with a growth rate of 8.69%. The degree centrality exceeded the average value, and centrality position was greatly improved. EU accession is conducive to strengthening the tourism economic ties between Croatia and other EU member states, and greatly enhancing Croatia’s position in the EU’s tourism economic network.

## 5. Analysis of the Spatial Network Effect of the EU Tourism Economy

### 5.1. Effect Analysis of the Complete Network Structure

Adopting the comprehensive index of Su (2009) [94], Formula (6) is used to measure the development level of the tourism economy.

(6) $$Z_{i}=\sqrt{P_{i}\times Q_{i}}$$

In the formula, *Z_i_* is the level of tourism economic development. *P_i_* is the industry scale index, which is calculated by international tourism receipts and international tourist arrivals. *Q_i_* is the industry quality index, which is represented by the proportion of international tourism receipts in GDP and the proportion of inbound tourists. The calculation method is as follows:

(7) $$P_{i}=\sqrt{m_{i}/\sum^{\;}m_{i}\times n_{i}/\sum^{\;}n_{i}}$$

(8) $$Q_{i}=\sqrt{m_{i}/GDP_{i}\times n_{i}/\sum^{\;}n_{i}}$$

In the formulas, *m_i_* and *n_i_* represent international tourism receipts and international tourist arrivals received by country *i*, and *GDP_i_* is the gross domestic product of country *i*.

On this basis, the coefficient of variation of the tourism economic development level is calculated. The EU tourism industry specialization index is calculated by dividing the international tourism receipts of a country by its total GDP.

Scholars have carried out research on the construction of regression models based on complete network structure effects. Liu et al. (2015) analyzed the spatial correlation network structure of energy consumption and its effect in China using energy intensity as the dependent variable and the complete network characteristic index as the explanatory variable [95]. Sun and Nie (2019) calculated the effects of the spatial correlation network of the provincial gray water footprint in China. Gray water footprint intensity was taken as the dependent variable, and the complete network characteristic index was taken as the explanatory variable [96]. Wang et al. (2017) used the coefficient of variation of the provincial tourism economic development level and the tourism industry specialization level as dependent variables, and the characteristic index of the complete network structure as the explanatory variable, to measure the complete network structure effect of China’s interprovincial tourism economic connection network [97]. On this basis, taking EU tourism industry specialization and the coefficient of variation of EU member states’ tourism development level as the two dependent variables and taking network density, connectedness, graph hierarchy and graph efficiency as the four explanatory variables, regression models are constructed. The natural logarithms of the dependent and explanatory variables are used in the ordinary least square regressions. The regression results are shown in Table 5.

#### 5.1.1. Effect of the Complete Network Structure on Tourism Industry Specialization

The regression results in Table 5 show that the regression coefficients of network density, connectedness, graph hierarchy and graph efficiency are 1.6419, −1.4153, 0.0972, and 2.2175, respectively, but only network density reaches the 10% significance level. This result shows that improvements in network density can effectively improve the level of tourism specialization and increase the tourism industry’s influence on GDP. The increase in network density means an increase in the number of network links, the strengthening of tourism economy ties between EU member states, and the promotion of the overall specialization level of the tourism industry. 

#### 5.1.2. Effect of the Complete Network Structure on the Tourism Economic Development Level

The regression results in Table 5 show that the regression coefficients of network density, connectedness, graph hierarchy and graph efficiency are 0.4092, −0.6490, 0.0166 and 1.2423, respectively, but only connectedness and graph efficiency reach the 5% significance level. This result indicates that improvements in connectedness and decreases in graph efficiency can significantly reduce the differences in the EU tourism economy and improve the spatial equity of tourism economic development. The reasons for this result are as follows: the increase in connectedness indicates that, as the total number of network connections increases, it promotes the allocation of tourism resources and market sharing among member states, deepens EU tourism economic cooperation, improves the level of tourism economic development of various countries, and effectively suppresses the spatial difference and polarization trend in the tourism economic development level. A decrease in graph efficiency means an increase in effective connections in the network and an increasingly close relationship in tourism economic development among nodes, which effectively reduces the monopoly held by a few nodes on tourism resources, the tourism market and tourism talent, improves the effectiveness of resource allocation in the tourism market and improves the stability of the network. The structure of the EU tourism economic connection network is constantly being strengthened, the differences in tourism economic development levels among member states are constantly narrowing, and the balance and spatial equity are constantly improving.

### 5.2. Effect Analysis of the Ego Network Structure

Some scholars have conducted research on regression models of the ego network structure effect. Liu et al. (2015) analyzed the complete network effect of the spatial correlation of China’s provincial energy consumption using provincial energy intensity as the dependent variable and the provincial ego network characteristic index as the explanatory variable [95]. Sun and Nie (2019) calculated the ego network effect of China’s provincial gray water footprint spatial association network, with provincial gray water footprint intensity as the dependent variable and the provincial ego network characteristic index as the explanatory variable [96]. Wang et al. (2017) used provincial tourism industry specialization as the dependent variable, the provincial ego network structure index as the explanatory variable, and tourism reception capacity, tourism human resources and accessibility indicators as control variables to measure the complete network structure effect of China’s provincial tourism economic connection network [97]. On this basis, the regression models take the tourism industry specialization of 28 EU member states from 1995 to 2018 as the dependent variable and take degree centrality, betweenness centrality and closeness centrality as explanatory variables, controlling for year and EU membership (if a country is an EU member in a certain year, the dummy variable is 1, otherwise it is 0). The natural logarithms of dependent variables and explanatory variables are used in regressions using panel data. The results of the fixed effect model, in Table 6, show that the regression coefficients of degree centrality and betweenness centrality are positive and reach the 1% significance level. The regression coefficient of closeness centrality is negative and reaches the 10% significance level. This shows that the centrality of each country in the tourism economic network plays a significant role in promoting the specialization level of the tourism industry.

The regression coefficient of degree centrality is 0.3129, which reaches the 1% significance level. The result shows that, the more extensive the association is between each country and other countries in the EU tourism economic connection network, the more conducive it is to improving network density and association, reducing graph hierarchy and graph efficiency, and enhancing the impact of the complete network on the countries. Countries’ profits from overall tourism economic development can be used to improve their tourism industry specialization level. Nodes with low degree centrality and high tourism specialization, such as Bulgaria, Slovenia, Luxembourg, Portugal, Estonia, Malta and Cyprus, should strengthen their tourism links with other countries, improve their degree centrality, and effectively improve the tourism industry specialization level.

The regression coefficient of betweenness centrality is 0.0331, which reaches the 1% significance level. In the tourism economic network, by relying on comparative advantages and control of the complete network, the countries with higher betweenness centrality guide the direction and quantity of tourist flows in other countries, strengthen the spatial spillover effect on other nodes, and promote improvement in the specialization level of the tourism industry in each node of the tourism economic network. Countries with low betweenness centrality and high tourism specialization, such as Croatia, Greece, Hungary, Estonia and Slovenia, should improve their status in the EU tourism economic network and strengthen their tourism economic ties with other countries to improve their tourism industry specialization level.

At the same time, countries with high degree centrality, betweenness centrality and closeness centrality but low specialization levels, such as Germany, France, Italy, Austria, Poland and Belgium, should give full play to the advantages of their central position in the network and promote the development of tourism in other countries while improving their tourism specialization level. Countries with high degree centrality and closeness centrality but low betweenness centrality, such as the United Kingdom, Netherlands, Spain and the Czech Republic, should strengthen their tourism links with other countries, improve betweenness centrality and enhance their tourism specialization level.

## 6. Conclusions and Implications

### 6.1. Conclusions

Based on the tourism economic data of 28 EU member states from 1995 to 2018, this paper uses the modified tourism economic gravity model to measure the tourism economic strength of countries, constructs a spatial association network, and uses the social network method to measure the structure and effect of the EU tourism economic network. The main conclusions are as follows.

(1) EU tourism economic associations show a trend of continuous growth, with France, the United Kingdom, Germany, Austria and Italy ranked at the forefront for tourism growth and Luxembourg, Lithuania, Latvia, Malta and Cyprus ranked at the bottom of the list.

(2) The complete network structure characteristics show an increasing annual trend in network density, which means that the tourism economic ties between EU member states are growing increasingly close. The degree of connectedness is increasing annually, the effect of spatial network connectivity is improving, and there are spatial correlation and spatial spillover effects in the tourism economy. Graph hierarchy and graph efficiency are decreasing annually, the hierarchical system is weakening, spatial correlation is increasing, and network stability is increasing.

(3) Ego networks have some structural characteristics. (1) The degree centrality of all member states shows a significant growth trend. Germany, France, Italy, Austria and the United Kingdom are firmly in the top five and in the central position of the network. Estonia, Romania, Malta, Latvia and Lithuania are at the bottom of the network ranking and are in a subordinate position. The kernel density estimation of degree centrality shows that a relatively small number of countries have more tourism economic ties, but the number is increasing with the passage of time; thus, a relatively large number of countries have fewer tourism economic ties, but this number is gradually decreasing with the passage of time. The centralization of the graph is increasing, and network concentration is increasing. (2) Most countries have improved their betweenness centrality. Germany, Italy, Sweden, Austria and France have strong betweenness centrality, which indicates that these countries play a controlling and dominating role in the network. The betweenness centrality of 11 countries, including Denmark, is zero, which indicates that they do not have the ability to control resources. (3) Germany, France, Italy, Britain and Austria are the top five countries for closeness centrality, and they are in the center of the network and hold great influence. Finland, Malta, Lithuania, Latvia and Cyprus are at the periphery of the network.

(4) The results of the core periphery analysis show that the core areas are mainly concentrated in Western Europe, Southern Europe, Mediterranean land countries and the neighboring countries of Central Europe, while the periphery areas are mainly concentrated in Eastern Europe, Northern Europe and Mediterranean island countries. The network connection density of core areas, peripheral areas, and transitional zones between core areas and peripheral areas shows an overall growth trend, which is promoting the development of the complete network connectedness of the EU tourism economy.

(5) The results of the network structure effect analysis show that improvement in network density can effectively improve the level of tourism specialization. The complete network structure has a significant impact on the tourism economic development level. The results of the network structure effect analysis show that improvement in connectedness and decrease in graph efficiency can significantly narrow the differences in the EU tourism economic development level and improve the spatial equity of tourism economic development. At the same time, improvement in degree centrality and betweenness centrality in the ego network plays a significant role in promoting the level of tourism industry specialization.

### 6.2. Implications

(1) Optimize the structure of the tourism economic network and improve the level of regional tourism integration. EU tourism economic attribute data and association data are basically consistent. In terms of international tourist arrivals and international tourism receipts, France, Spain, Italy, Germany and the United Kingdom were the top five countries in 2018. The social network analysis results show that Germany, France, Italy, Austria and Poland are the top five countries in terms of degree centrality. Germany, Italy, Sweden, Austria and France are the top five countries in terms of betweenness centrality. Germany, Italy, France, Austria and the United Kingdom are the top five countries in terms of closeness centrality. In the process of promoting the development of EU regional tourism integration, it is important to pay attention not only to attribute data but also to the network structure effect of tourism economic links. It is necessary to give full play to the central and intermediary role of Germany, France, Italy, Austria, the United Kingdom, Poland, and Sweden, to optimize the network structure of EU tourism economic associations and to engage in the mutual promotion of regional tourism integration and network structure. EU regional tourism integration will benefit from the implementation of the policies of the single market, Euro and Schengen Agreement. Among them, the impact of EU development on tourism flow includes a tourism creation effect, tourism transfer effect, tourism spillover effect and tourism investment effect. The impact of the euro on tourism flows includes avoiding effects from exchange and administrative costs, enhancing the tourism market potential of the euro area, strengthening tourism cooperation in the euro area and improving the international competitiveness of the tourism enterprise. The promotion mechanism of the Schengen visa policy on tourism flow stimulates the business tourism market and the visiting friends and relatives leisure tourism market. The “same country effect” of the Schengen visa promotes the establishment of a single tourism market. The Schengen visa promotes an increase in tourism flows through the spatial spillover effect of countries, and a change in the list of positive and negative countries in the Schengen Agreement increases tourism demand. Regional tourism integration in the EU strengthens tourism economic connections and optimizes the network structure. The optimization of the tourism economic connection network structure, in turn, promotes improvement in the tourism integration level.

**Figure 6:**
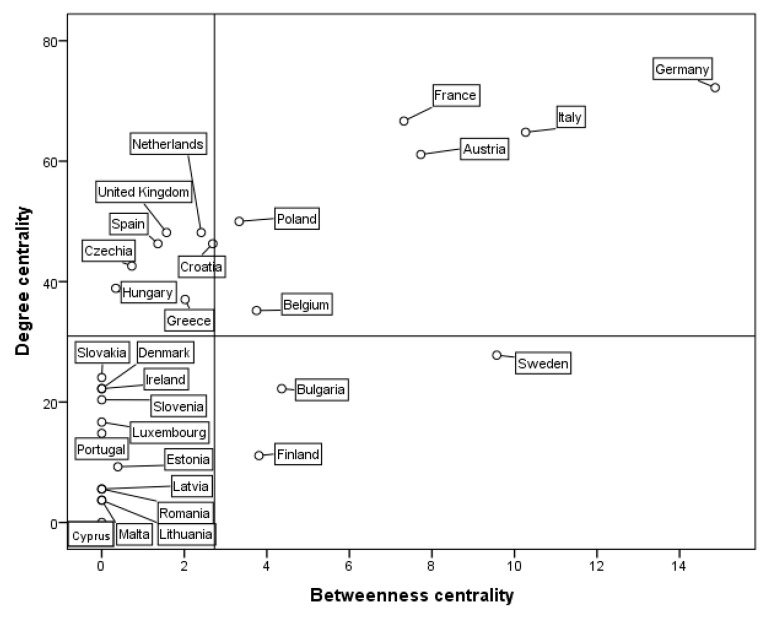
Two-dimensional distribution map of the centrality of EU member states (2018).

Based on the degree centrality and betweenness centrality index of EU member states in 2018, a two-dimensional distribution map is constructed, with the X-axis as betweenness centrality and the y-axis as degree centrality. The mean values of degree centrality and betweenness centrality are used as segmentation points of the x-axis and y-axis (2.73, 30.95), dividing the two-dimensional distribution map into four quadrants (in Figure 6).

In the first quadrant are countries with high degree centrality and betweenness centrality, including Germany, Italy, France, Austria, Poland and Belgium. They are in the center of the network, with strong power and strong control over other countries. They play a central role in the network, radiate toward other countries, strengthen the intermediary role of other nodes, and enhance network links. In the second quadrant are countries with high degree centrality and low betweenness centrality, including the United Kingdom, the Netherlands, Spain, Hungary, the Czech Republic, Greece and Croatia. They play a central role in the network but have a low intermediary effect on other countries. They should give full play to the advantages of their central position and radiate toward other countries. In the third quadrant, there are countries with low degree centrality and betweenness centrality. Among them, the betweenness centrality of Slovakia, Ireland, Denmark, Slovenia, Luxemburg, Portugal, Romania, Latvia, Lithuania, Malta and Cyprus is 0, and the betweenness centrality of Estonia is 0.39. These countries are not in the center of the network and have no control over other countries. They should take the initiative to strengthen their tourism economic associations with other EU countries and improve their network centrality. In the fourth quadrant are countries with low degree centrality and high betweenness centrality, including Sweden, Bulgaria and Finland. Although these countries are not in the center of the network, they have strong control over other countries and have many structural holes. They should give full play to their intermediary and bridge roles in the network and improve their degree centrality.

(2) According to the structural characteristics of the core peripheral network, different regions should play different roles. Tourism resources, economic development level and spatial distance jointly affect the core peripheral structure of the EU tourism economic network. Countries in the network core are mainly located in the core area of the European continent, have rich tourism resources and solid economic foundations, allowing them to provide strong support for the development of tourism. On the demand side, high per capita disposable income determines the huge potential of tourism demand in these markets and makes these countries the main source of tourists in the world. On the supply side, the developed economic level in these countries is conducive to the development of tourism projects, the cultivation of tourism talent, the improvement of the level of tourism facilities, and the provision of high-quality tourism products and experiences to meet the needs of tourists. The countries in the network periphery are located far from the tourism market center of the European continent. With this increase in spatial distance, these countries are gradually moving away from the origin in the core area of the European continent, and their tourism economic connection strength is gradually reducing, creating peripheral areas. The density of countries in the core area is high, and the economic connection density is also high, which makes it an important region for the EU tourism economy. On the one hand, the core area countries are rich in tourism resources and convenient for international aviation, which is conducive to the entry and exit of international tourists. The core area countries should give full play to their gateway role to drive the tourism development of peripheral countries. On the other hand, the core area also provides important tourists for the peripheral areas and realizes close interaction with the peripheral area countries.

(3) The complete network structure and ego network structure should play a role in promoting the level of tourism industry specialization. It is important to increase the number of tourism economic networks between countries, create more links, increase network density and relevance, and improve the spatial equity of tourism economic development. Countries with low centrality and high specialization should actively establish extensive contacts with other EU member states to improve the level of tourism industry specialization. At the same time, it is important to promote the transformation of some countries from a one-way link to a two-way link and realize their transformation from subordinate status to equal status.

In the future, we will research the driving factors for the evolution of the spatial network structure of EU tourism economy connection. The driving factors include economic development, openness, trade links, geographical proximity and accession time to the EU (if a country is an EU member in a certain year, the dummy variable is 1, otherwise it is 0), etc. We will construct a difference matrix and conduct a quadratic assignment procedure (QAP) to analyze and measure the effect of driving factors on the evolution of the EU’s tourism economy network structure.

## Figures and Tables

**Table 1:** Tourism economic connections in the EU from 1995 to 2018.

Nodes (Countries)	1995			2002			2009			2018		
	Tourism Economic Connection	Percentage (%)	Rank	Tourism Economic Connection	Percentage (%)	Rank	Tourism Economic Connection	Percentage (%)	Rank	Tourism Economic Connection	Percentage (%)	Rank
United Kingdom	1084.47	23.58	1	1478.52	21.88	1	2324.60	19.06	2	3803.09	16.29	2
France	996.51	21.67	2	1412.76	20.91	2	2361.92	19.37	1	4234.59	18.14	1
Germany	443.20	9.64	3	561.61	8.31	3	1200.96	9.85	4	2251.32	9.64	4
Italy	330.46	7.19	4	464.54	6.87	6	803.96	6.59	5	1591.39	6.82	5
Austria	304.63	6.62	5	539.85	7.99	4	1317.83	10.81	3	2859.41	12.25	3
Netherlands	271.44	5.90	6	520.73	7.71	5	629.49	5.16	7	1362.10	5.84	7
Slovakia	251.66	5.47	7	279.20	4.13	8	612.37	5.02	8	1127.91	4.83	8
Spain	236.27	5.14	8	402.56	5.96	7	690.83	5.67	6	1483.76	6.36	6
Poland	120.18	2.61	9	104.59	1.55	13	186.51	1.53	13	445.52	1.91	13
Hungary	106.94	2.33	10	129.67	1.92	11	256.12	2.10	11	545.44	2.34	12
the Czech Republic	104.94	2.28	11	134.18	1.99	10	310.25	2.54	10	549.67	2.35	11
Belgium	99.35	2.16	12	230.30	3.41	9	368.65	3.02	9	632.91	2.71	9
Denmark	51.92	1.13	13	71.21	1.05	14	166.82	1.37	14	179.95	0.77	20
Greece	35.49	0.77	14	66.04	0.98	15	118.75	0.97	15	252.77	1.08	14
Croatia	31.61	0.69	15	111.44	1.65	12	252.31	2.07	12	631.33	2.70	10
Ireland	29.85	0.65	16	60.79	0.90	16	103.37	0.85	16	203.25	0.87	18
Sweden	25.33	0.55	17	50.24	0.74	17	94.56	0.78	17	205.19	0.88	17
Finland	21.12	0.46	18	28.40	0.42	19	85.37	0.70	18	186.12	0.80	19
Bulgaria	14.14	0.31	19	23.74	0.35	20	72.48	0.59	20	136.50	0.58	21
Portugal	13.99	0.30	20	32.14	0.48	18	76.47	0.63	19	247.59	1.06	15
Slovenia	7.39	0.16	21	16.06	0.24	22	70.37	0.58	21	230.72	0.99	16
Romania	6.94	0.15	22	5.53	0.08	24	31.36	0.26	22	47.72	0.20	23
Luxembourg	5.04	0.11	23	9.44	0.14	23	17.66	0.14	24	23.70	0.10	24
Estonia	2.19	0.05	24	16.22	0.24	21	24.26	0.20	23	72.16	0.31	22
Malta	1.48	0.03	25	1.58	0.02	27	2.07	0.02	27	5.58	0.02	27
Lithuania	1.12	0.02	26	3.16	0.05	25	6.10	0.05	26	17.29	0.07	25
Cyprus	0.71	0.02	27	1.22	0.02	28	1.52	0.01	28	3.07	0.01	28
Latvia	0.44	0.01	28	2.17	0.03	26	6.35	0.05	25	12.15	0.05	26
max	1084.47	23.58		1478.52	21.88		2361.92	19.37		4234.59	18.14	
min	0.44	0.01		1.22	0.02		1.52	0.01		3.07	0.01	
mean	164.24	3.57		241.35	3.57		435.47	3.57		833.65	3.57	
total	4598.81	100		6757.89	100		12193.31	100		23342.20	100	

Note: Calculated by the authors.

**Table 2:** Degree centrality of the tourism economic network in the EU from 1995 to 2018.

Countries	Out Degree				Countries	in Degree				Countries	Degree Centrality			
	1995	2002	2009	2018		1995	2002	2009	2018		1995	2002	2009	2018
Germany	15	17	19	23	Austria	15	14	16	17	Germany	46.30	53.70	59.26	72.22
Italy	18	16	20	22	France	11	14	16	16	France	51.85	59.26	66.67	66.67
France	17	18	20	20	Germany	10	12	13	16	Italy	50.00	46.30	57.41	64.81
the UK	13	15	16	18	Croatia	5	12	14	15	Austria	48.15	48.15	55.56	61.11
Spain	10	12	15	17	Italy	9	9	11	13	Poland	35.19	25.93	33.33	50.00
Austria	11	12	14	16	Poland	11	9	10	13	the UK	37.04	40.74	42.59	48.15
Netherlands	8	12	12	14	the Czech Republic	6	7	12	12	Netherlands	31.48	37.04	38.89	48.15
Poland	8	5	8	14	Netherlands	9	8	9	12	Croatia	12.96	29.63	40.74	46.30
the Czech Republic	4	5	10	11	Belgium	8	8	11	11	Spain	27.78	33.33	38.89	46.30
Hungary	5	6	9	10	Hungary	5	6	8	11	the Czech Republic	18.52	22.22	40.74	42.59
Croatia	2	4	8	10	Greece	2	7	7	11	Hungary	18.52	22.22	31.48	38.89
Greece	2	5	6	9	Slovenia	4	5	7	9	Greece	7.41	22.22	24.07	37.04
Sweden	2	3	5	9	the UK	7	7	7	8	Belgium	24.07	25.93	31.48	35.19
Belgium	5	6	6	8	Luxembourg	6	6	7	8	Sweden	5.56	9.26	16.67	27.78
Ireland	2	3	4	6	Slovakia	3	3	7	8	Slovakia	7.41	11.11	20.37	24.07
Denmark	5	4	8	5	Spain	5	6	6	8	Bulgaria	1.85	5.56	18.52	22.22
Slovakia	1	3	4	5	Denmark	3	4	9	7	Denmark	14.81	14.81	31.48	22.22
Bulgaria	0	1	4	5	Bulgaria	1	2	6	7	Ireland	9.26	12.96	16.67	22.22
Portugal	1	2	2	3	Ireland	3	4	5	6	Slovenia	9.26	11.11	16.67	20.37
Finland	1	1	1	3	Sweden	1	2	4	6	Luxembourg	11.11	11.11	14.81	16.67
Slovenia	1	1	2	2	Portugal	4	3	4	5	Portugal	9.26	9.26	11.11	14.81
Estonia	0	1	1	2	Finland	0	2	2	3	Finland	1.85	5.56	5.56	11.11
Luxembourg	0	0	1	1	Estonia	1	1	1	3	Estonia	1.85	3.70	3.70	9.26
Romania	0	0	1	1	Latvia	0	0	0	3	Latvia	0.00	0.00	0.00	5.56
Cyprus	0	0	0	0	Romania	1	0	3	2	Romania	1.85	0.00	7.41	5.56
Latvia	0	0	0	0	Malta	1	1	1	2	Lithuania	0.00	0.00	0.00	3.70
Lithuania	0	0	0	0	Lithuania	0	0	0	2	Malta	1.85	1.85	1.85	3.70
Malta	0	0	0	0	Cyprus	0	0	0	0	Cyprus	0.00	0.00	0.00	0.00
mean	4.68	5.43	7	8.36	mean	4.68	5.43	7	8.36	mean	17.33	20.11	25.93	30.95
centrality (%)	51.17%	48.29%	49.93%	56.24%	centrality	39.64%	32.92%	34.57%	33.2%	centrality (%)	37.18%	42.17%	43.88%	44.44%

Note: Countries are ranked according to the 2018 calculation.

**Table 3:** Betweenness centrality and closeness centrality of the tourism economic network in the EU from 1995 to 2018.

Countries	Betweenness Centrality				Countries	Closeness Centrality			
	1995	2002	2009	2018		1995	2002	2009	2018
Germany	7.604	8.78	7.268	14.855	Germany	15.976	19.014	23.684	47.368
Italy	12.903	9.065	8.027	10.271	Italy	16.265	18.621	23.478	46.552
Sweden	0	5.413	5.983	9.568	France	16.168	19.149	23.894	45
Austria	12.831	7.213	9.188	7.734	the UK	15.789	18.493	23.077	43.548
France	8.984	15.045	13.449	7.316	Austria	15.976	18.493	22.689	43.548
Bulgaria	0	0	3.678	4.358	Spain	15.517	18.121	22.5	42.188
Finland	0	2.849	3.134	3.806	Poland	15.698	17.881	22.131	42.188
Belgium	0.332	0.347	3.742	3.751	Netherlands	15.517	18.121	21.951	41.538
Poland	1.93	0.14	0.491	3.327	Croatia	15.169	18.121	22.314	40.909
Croatia	0.103	4.009	3.619	2.689	the Czech Republic	15.169	17.647	22.314	39.706
Netherlands	0.819	1.426	1.551	2.406	Belgium	15.429	17.763	21.774	39.13
Greece	0	3.986	1.424	2.023	Greece	14.835	17.647	21.429	39.13
the UK	2.473	1.936	2.373	1.569	Hungary	15.254	17.532	21.429	39.13
Spain	3.245	1.347	1.195	1.362	Sweden	14.286	17.197	21.094	39.13
the Czech Republic	0	0.018	1.144	0.732	Denmark	14.917	17.089	22.131	37.5
Estonia	0	0	0	0.387	Slovenia	14.917	13.308	21.094	37.5
Hungary	0.199	1.246	0.566	0.34	Luxembourg	15.169	17.419	21.094	36.986
Denmark	0	0	1.117	0	Slovakia	14.516	16.77	21.094	36.986
Cyprus	0	0	0	0	Bulgaria	14.439	16.265	21.094	36.986
Ireland	0	0	0	0	Ireland	14.516	16.981	20.611	36.486
Latvian	0	0	0	0	Portugal	14.835	16.77	20.611	35.526
Latvian	0	0	0	0	Finland	3.704	15.254	18.121	34.615
Luxembourg	0	0	0	0	Estonia	3.704	13.568	15.698	34.615
Malta	0	0	0	0	Latvian	—	—	—	33.333
Portugal	0	0	0	0	Romania	14.439	—	19.853	32.927
Romania	0	0	0	0	Malta	14.439	16.168	19.565	32.927
Slovakia	0	0	0	0	Latvian	—	—	—	29.348
Slovenia	0	0	0	0	Cyprus	—	—	—	—
mean	1.84	2.24	2.43	2.73					
centrality (%)	11.48	13.28	11.43	12.57					

Note: Countries are ranked according to the 2018 calculation.

**Table 4:** Development process and grouping of EU countries.

	State	EU Member Country	Euro Area Member	Schengen Area Member
Northern Europe	Denmark	1973		2001
	Finland	1995	2002	2001
	Ireland	1973	2002	
	Sweden	1995		2001
	Norway			2001
	Iceland			2001
	United Kingdom	1973		
Western Europe	Austria	1995	2002	1997
	Belgium	1958	2002	1995
	France	1958	2002	1995
	Germany	1958	2002	1995
	Switzerland			2008
	Liechtenstein			2011
	Luxembourg	1958	2002	1995
	Netherlands	1958	2002	1995
Central/Eastern Europe	Bulgaria	2007		
	Czech Republic	2004		2007
	Estonia	2004	2011	2007
	Hungary	2004		2007
	Latvia	2004	2014	2007
	Lithuania	2004	2015	2007
	Poland	2004		2007
	Romania	2007		
	Slovakia	2004	2009	2007
Southern/Medit. Europe	Croatia	2013		
	Cyprus	2004	2008	
	Greece	1981	2002	2000
	Italy	1958	2002	1997
	Malta	2004	2008	2007
	Portugal	1986	2002	1995
	Slovenia	2004	2007	2007
	Spain	1986	2002	1995

Sources: World Tourism Organization (2018), European Union Tourism Trends, United Nations World Tourism Organization, Madrid.

**Table 5:** Effect of the complete network structure.

Variables	Tourism Industry Specialization	Coefficient of Variation of Tourism Economic Development Level
	(1)	(2)
constant	3.9863 **	0.8538
Network Density	1.6419 *	0.4092
Connectedness	−1.4153	−0.6490 **
Graph Hierarchy	0.0972	0.0166
Graph Efficiency	2.2175	1.2423 **
R^2^	0.4012	0.9316

Note: **, and * represent significance levels of 5% and 10%, respectively.

**Table 6:** Effect of the ego network structure.

Variables	Tourism Industry Specialization
Constant	0.7400 ***
Degree centrality	0.3129 ***
Betweenness centrality	0.0331 ***
Closeness centrality	−0.0384 *
R^2^	0.3068
Number of observations	672

Note: *** and * represent significance levels of 1% and 10%, respectively.

## Data Availability

The data presented in this study are available on request from the corresponding author.
